# Psychometric Properties of the Suicide Stroop Task in a Chinese College Population

**DOI:** 10.3389/fpsyg.2021.586391

**Published:** 2021-02-26

**Authors:** Lu Niu, Xia Feng, Zhouxin Jia, Yu Yu, Liang Zhou

**Affiliations:** ^1^Department of Social Medicine and Health Management, Xiangya School of Public Health, Central South University, Changsha, China; ^2^The Affiliated Brain Hospital of Guangzhou Medical University, Guangzhou, China; ^3^School of Economic and Management, Guangzhou University of Chinese Medicine, Guangzhou, China; ^4^Hospital Evaluation Office, Xiangya Hospital, Central South University, Changsha, China

**Keywords:** suicide, suicide stroop task, attentional bias, reliability, validity

## Abstract

**Objective:**

This study aimed to test the psychometric properties of the suicide stroop task in a Chinese college population.

**Methods:**

College students (*n* = 121) who were in the 1st–4th grade, fluent in Chinese, and without color blindness were recruited from a university in Guangzhou, China from September to December 2019. Participants were administered the suicide stroop task at baseline and 1-month follow-up.

**Results:**

The suicide stroop task showed excellent internal reliability (Cronbach’s α ranged from 0.940 to 0.953). However, the suicide stroop task did not reveal suicide-related attentional biases among current suicide ideators and was not significantly associated with the severity of suicidal ideation, depression, hopelessness, nor anhedonia (all *p* values > 0.05), indicating a lack of concurrent validity for the task. Additionally, the two-time data of interference scores could not generate intraclass correlation coefficients (ICCs) due to a negative average covariance among data, which indicated poor test–retest consistency for the task.

**Conclusion:**

The results of this study did not support the use of the suicide stroop task on the identification of suicidal risk among Chinese college students. It is crucial to assess the psychometric properties of behavioral measures rigorously as self-report measures before large applications in clinical and community settings.

## Introduction

Suicide is a major public health issue in young people, with suicide being the second leading cause of death in people between the ages of 15 and 29 years worldwide (Turecki and Brent, 2016). Additionally, suicide has received increasing attention among subgroups of these young people including college students. A meta-analysis showed that pooled prevalence estimates of lifetime suicidal ideation, plans, and attempt were 22.3, 6.1, and 3.2% among college students, and higher estimates were found in samples from Asia (Mortier et al., 2018). It is important to effectively identify people at risk for suicide behaviors to prevent fatal attempt, but the prediction of suicide continues to be a critical challenge (Franklin et al., 2017).

Currently, the screening of suicide risk commonly relies on self-report. However, self-report assessments are limited by the individuals’ willingness (e.g., to avoid hospitalization) and ability to report suicidal thoughts (i.e., not aware of suicidal thoughts/suicidal risk) (Glenn et al., 2019). Moreover, a systematic review found that the Beck Hopelessness Scale and the Beck Suicide Intent Scale, two commonly used self-report suicide risk scales, did not have sufficient evidence to support their use on predicting suicide in high-risk samples (Chan et al., 2016). Thus, it seems insufficient to identify suicide risk by self-report alone, and there are increasing arguments on the need of more objective tools on suicide risk determination.

According to the cognitive model of suicidal behavior, suicide-specific attentional bias leads to a fixation on suicide as the sole escape solution, and combined with a state of hopelessness, it would ultimately result in a suicide attempt (Wenzel and Beck, 2008). Previous research found that suicide-specific attentional bias is relevant to previous suicidal attempts in clinical samples (Williams and Broadbent, 1986; Becker et al., 1999; Cha et al., 2010). Specifically, the study conducted by Cha et al. (2010) suggested that suicide-specific attentional bias can be used as a potential behavioral marker to predict future suicide attempt. As these results were very promising, researchers in different countries tried to generalize the measure used in Cha et al.’s study (Cha et al., 2010), the suicide stroop task, into different samples including college students, patients with mood disorders, and community-based samples reporting past-month suicidal ideation (Chung and Jeglic, 2016; Richard-Devantoy et al., 2016; Cha et al., 2017). However, mixed findings were reported. Additionally, a systematic review of the existing seven studies found that the suicide stroop task had excellent internal reliability, but poor classification accuracy to classify suicide attempter from non-attempters (Wilson et al., 2019).

The validity of the suicide stroop task has not been tested in the Chinese context. In this current study, we made a Chinese-language adaption of the suicide stroop task and tested its internal reliability, concurrent validity, and test–retest reliability in Chinese college students. This study aimed to provide more evidence whether the suicide stroop task could be used in a community-based sample in which the majority would not report suicidal ideation and have never made a serious suicidal attempt before. Based on previous research, we hypothesized that (1) those who reported current suicidal ideation (current SI) would also have slower reaction times to suicide-related words than those without current suicidal ideation (non-ideator) and (2) the performance of the suicide stroop task would be significantly associated with suicidal ideation severity, depression, hopelessness, and anhedonia.

## Methods

### Participants and Procedures

College students who were in the 1st–4th grade, fluent in Chinese, and without color blindness were recruited from a university in Guangzhou, China from September to December 2019. Participants were recruited online (e.g., WeChat group). Interested participants were invited to a computer laboratory. All participants were asked to provide written informed consent and then to complete the baseline survey and the suicide stroop task. One month later, participants were invited to complete the retest survey and the suicide stroop task in the same laboratory.

This study was approved by the institutional review boards of the Affiliated Brain Hospital, Guangzhou Medical University. Written informed consent has been obtained from all participants.

### The Suicide Stroop Task

The suicide stroop task is a computer-based behavior task that uses response latencies of how quickly participants identify the color of different words presented on a computer screen. The test material and test conditions were replicated based on the methodology used in Cha et al. (2010). In this study, stimuli for the task were presented, and response latencies were recorded using E-prime 2.0 software.

After reading the instructions, participants were asked to complete eight practice trial, followed by 48 critical trials. Each trial started with a blank white screen for 4 s, followed by a centered “+” in red for 1 s, another blank screen for 1 s, and then the word either in blue or in red color; the words remained on the screen until either a blue or a red key was pressed.

During the critical trial, neutral [house (*fangwu*), paper (*baizhi*), and car (*qiche*)], positive [happy (*kaixin*), success (*chenggong*), and pleasure (*kuaile*)], negative [alone (*gudu*), rejected (*jujue*), and stupid (*yuchun*)], and suicide-related [funeral (*zangli*), dead (*siwang*), and suicide (*zisha*)] words in Chinese characters were presented. After discussion with psychologists, museum, and engine, which were used as neutral words by Cha et al. (2010), were replaced by house and car (in Chinese characters) based on the Chinese contexts in this study. Each of these words was presented four times in random order during the 48 critical trials. The interference score for each category was calculated by subtracting the mean response time (RT) for neutral words from the mean RT for positive, negative, or suicide-related words.

### Measures

#### Socio-Demographics

Socio-demographic information including age, gender, residence, single child or not, and relationship status was collected.

#### History of Suicidal Attempts

In this study, we used the introduction interview part of the Pathway to Suicidal Action Interview (PSAI) to collect data on previous suicidal behaviors. Approved and assisted by the first author of the PSAI [Millner, A.J. (Millner et al., 2017)], a panel of three bilingual public health researchers, who were also trained in psychiatry and suicide prevention, translated the original English version of the PSAI into simplified Chinese. For an action to be considered as a suicidal attempt, an individual must have had engaged in a potentially deadly behavior with some intention to die (Millner et al., 2017).

#### Current Suicidal Ideation

The Beck Sale for Suicidal Ideation (BSSI) (Beck et al., 1979) was used to assess the severity of suicidal ideation in the past week. Each item is rated on a 0–2-point scale, with higher scores reflecting more severe suicidal ideation. If one rated either item four or five with a score of one or greater, the person was considered as having current suicidal ideation.

#### Depression

The degree of depression was assessed by the Patient Health Questionnaire Depression Scale (PHQ-9) (Bian et al., 2009). It consists of nine items related to the diagnostic criteria of major depressive disorder based on the Diagnostic and Statistical Manual of Mental Disorders, Fourth Edition (DSM-IV). The total score ranges from 0 to 27, with higher scores indicating higher levels of depression.

#### Hopelessness

Hopelessness was measured by the 4-item Beck’s Hopelessness Scale (BHS-4) (Yip and Cheung, 2006; Ma et al., 2020). It consists of four items relevant to success, dark future, breaks, and faith. Item responses range from 1 (strongly agree) to 5 (strongly disagree). The possible score ranges from four to 20, and a higher score represents a higher level of hopelessness.

#### Anhedonia

Anhedonia was measured by the Snaith–Hamilton Pleasure Scale (SHAPS) (Snaith et al., 1995). It is a validated and reliable scale that was developed to assess the ability to experience pleasure in normally pleasurable activities in the past few days. It consists of 14 items, and each item is rated on a 4-point Likert format, ranging from 1 (strongly agree) to 4 (strongly disagree) (Hu et al., 2017). The total score ranges from 14 to 56, with higher scores indicating lower ability to experience pleasure.

### Statistical Analysis

Regarding the suicide stroop task, we included trials with correct responses in the analysis. For all participants, the rate of correct response was 97.7%, and the correct response rates for suicide-related (97.5%), negatively-valenced (97.0%), positive-valenced (97.9%), and neutral words (98.3%) did not significantly differ from one another (χ^2^ = 5.301, *p* = 0.151). Additionally, we eliminated trials with response latencies ±2 SD from each participant’s mean response latency.

Internal reliability was evaluated using the criterion of Cronbach’s alpha ≥ 0.70. Regarding concurrent validity, we firstly performed independent sample *t*-tests to assess the group differences in mean RTs or interference scores (suicide/negative/positive word RT–neutral word RT) for each valence word, and then we conducted Group × Valence (repeated measures analysis) ANOVAs. Group comparisons included current ideators vs. non-ideators. For the within-subjects factor, valence had four levels in mean RT analyses (i.e., suicide-related, negative, positive, and neutral) and three levels in interference scores (i.e., suicide-related, negative, and positive). Additionally, Pearson correlation analysis was used to evaluate the correlations between suicide stroop task performance (mean RTs and interference scores) and the severity of current suicidal ideation, depression, hopelessness, and anhedonia. Test–retest reliability was assessed by the paired-sample *t*-test and intraclass correlation coefficients (ICCs). All analyses were performed by SPSS version 23.0 (SPSS Inc., Chicago, IL, United States). The level of significance was set at 0.05.

## Results

### Demographic Characteristics

As presented in Table 1, a total of 121 college students participated in this study. Among them, 62.0% were female, and the mean age was 19.0 years (SD = 4.1). There were 3.3% of participants reporting previous suicidal attempts and 28.9% reporting current suicidal ideation. One month after baseline, 103 students (85%) completed the retest. Except for previous suicidal attempts, no significant differences were found in socio-demographic or psychosocial characteristics at baseline between lost samples and those who finished the retest (Table 1).

**TABLE 1:** Sample characteristics and attrition analysis.

	**Baseline (*n* = 121)**	**1-month retest**			
		**Loss (*n* = 18)**	**Non-loss (*n* = 103)**	**χ^2^/*t***	***p***
Age (mean ± SD)	19.0 ± 4.1	19.7 ± 5.0	18.9 ± 4.0	0.729	0.468
Gender				0.007	0.934
Male	46 (38.0)	7 (38.9)	39 (37.9)		
Female	75 (62.0)	11 (61.1)	64 (62.1)		
Residence				0.476	0.490
Urban	83 (68.6)	13 (72.2)	70 (68.0)		
Rural	38 (31.4)	5 (27.8)	33 (32.0)		
Single child				0.129	0.719
Yes	45 (37.2)	8 (44.4)	37 (35.9)		
No	76 (62.8)	10 (55.6)	66 (64.1)		
Relationship status				1.235	0.266
Single	93 (76.9)	12 (66.7)	81 (78.6)		
In a relationship	28 (23.1)	6 (33.3)	22 (21.4)		
Previous suicidal attempt				4.030	0.045
No	117 (96.7)	16 (88.9)	101 (98.1)		
Yes	4 (3.3)	2 (11.1)	2 (1.9)		
Current suicidal ideation				0.462	0.497
No	86 (71.1)	14 (77.8)	72 (6939)		
Yes	35 (28.9)	4 (22.2)	31 (30.1)		
Severity of SI (mean ± SD)	2.5 ± 4.2	1.7 ± 3.3	2.7 ± 4.4	0.907	0.366
Depression (mean ± SD)	5.0 ± 3.5	4.1 ± 3.7	5.2 ± 3.5	1.227	0.222
Hopelessness (mean ± SD)	7.6 ± 2.3	7.9 ± 1.8	7.6 ± 2.3	0.526	0.600
Anhedonia (mean ± SD)	24.1 ± 5.3	22.6 ± 4.7	24.3 ± 5.4	1.242	0.217

### Internal Reliability

The mean RTs for each valence word demonstrated excellent internal reliability (Cronbach’s α ranged from 0.940 to 0.953).

### Concurrent Validity

Across the sample, a significant difference was found from the mean RT for suicide-related words, *M* = 513.03 (SD = 142.39 ms); negative valenced words, *M* = 513.33 (SD = 151.64 ms); positive valenced words, *M* = 499.55 (SD = 144.47 ms); and neutral words, *M* = 507.55 (SD = 151.70 ms), *F* = 5.139, *p* = 0.025. A least significant difference (LSD) analysis was conducted for multiple comparisons. The results of LSD indicated that the mean RT for suicide-related words and negative valenced words was significantly longer than the mean RT for positive valence words (*d*s = −13.486, −13.782, *p*s < 0.05).

As shown in Table 2, the results of independent sample *t*-tests revealed that no group difference in mean RTs or interference scores for each valence word was related to current suicidal ideation (*t* = 0.410–1.012, *p* = 0.314–0.683). Group × Valence interactions (repeated measures analysis) were also not significant when testing mean RTs or interference scores for two-group comparison (current SI vs. non-ideators, *F* = 0.795, *p* = 0.374).

**TABLE 2:** Comparing the suicide stroop performance across groups of suicidal behaviors (*n* = 121).

Score*	**Overall (*n* = 121)**	**Current suicidal ideation**			
		**Yes (*n* = 35)**	**No (*n* = 86)**	***t***	***p***
Mean RT_Sui_	513.03 ± 142.39	530.00 ± 163.72	506.13 ± 133.17	0.835	0.405
Mean RT_Neg_	513.33 ± 151.64	529.20 ± 177.64	506.87 ± 140.33	0.733	0.465
Mean RT_Pos_	499.55 ± 144.47	518.46 ± 170.60	491.85 ± 132.73	0.918	0.361
Mean RT_Neu_	507.55 ± 151.70	529.42 ± 176.81	498.65 ± 140.38	1.012	0.314
Interference_Sui_	5.48 ± 42.23	0.58 ± 44.72	7.48 ± 41.27	0.814	0.417
Interference_Neg_	5.78 ± 44.31	−0.22 ± 32.66	8.22 ± 48.21	0.950	0.344
Interference_Pos_	−8.00 ± 50.58	−10.97 ± 41.03	−6.80 ± 54.16	0.410	0.683

All suicide stroop score means and standard deviations are reported in milliseconds (ms). *Mean RT, mean response time; interference, suicide/negative/positive word RT–neutral word RT.

As shown in Table 3, the results of Pearson correlation analysis showed that the interference score for each valence word was not significantly associated with the scores of suicidal ideation severity, depression, hopelessness, or anhedonia (*r*s = −0.094–0.085, *p*s > 0.05).

**TABLE 3:** Partial correlations of the suicide stroop performance and other psychosocial variables (*n* = 121).

	**1**	**2**	**3**	**4**	**5**	**6**	**7**
Interference_Sui_	1.000	–	–	–	–	–	–
Interference_Neg_	0.663**	1.000	–	–	–	–	–
Interference_Pos_	0.402**	0.299**	1.000	–	–	–	–
Severity of SI	–0.082	–0.094	–0.022	1.000	–	–	–
Depression	0.011	0.024	0.051	0.307**	1.000	–	–
Hopelessness	–0.010	0.038	0.085	0.390**	0.321**	1.000	–
Anhedonia	0.035	–0.003	–0.015	0.225*	0.325**	0.363**	1.000

All suicide stroop score means and standard deviations are reported in milliseconds (ms). Interference, suicide/negative/positive word RT–neutral word RT. *< 0.05; **< 0.01.

### Test–Retest Reliability

As shown in Table 4, the paired-sample *t*-test showed no significant differences for mean RTs or interference scores of each valence word between baseline and retest. However, the two-time data of interference scores could not generate ICC values due to a negative average covariance among data, which violated reliability model assumptions.

**TABLE 4:** Comparing the suicide stroop performance and other psychosocial variables between baseline and retest (*n* = 103).

	**Baseline**	**Retest**	***t***	***p***	ICC*	***p***
Mean RT_Sui_	516.36 ± 147.35	507.78 ± 124.34	0.856	0.394	0.838	0.000
Mean RT_Neg_	517.07 ± 157.07	502.37 ± 119.66	1.445	0.151	0.842	0.000
Mean RT_Pos_	504.21 ± 151.28	501.66 ± 126.56	0.227	0.821	0.799	0.000
Mean RT_Neu_	511.62 ± 158.19	497.06 ± 106.35	1.394	0.166	0.817	0.000
Interference_Sui_	4.74 ± 42.85	10.72 ± 47.01	0.842	0.402	–0.801	0.998
Interference_Neg_	5.45 ± 45.64	5.30 ± 43.74	0.023	0.982	–0.081	0.652
Interference_Pos_	−7.41 ± 52.37	4.60 ± 50.03	1.530	0.129	–0.533	0.984
Severity of SI	2.65 ± 4.38	2.43 ± 4.37	0.736	0.464	0.859	0.000
Depression	5.16 ± 3.48	4.67 ± 3.45	1.473	0.144	0.696	0.000
Hopelessness	7.58 ± 2.35	7.75 ± 2.45	0.796	0.428	0.762	0.000
Anhedonia	24.30 ± 5.42	23.70 ± 5.90	1.343	0.182	0.808	0.000

All suicide stroop score means and standard deviations are reported in milliseconds (ms). Mean RT, mean response time; interference, suicide/negative/positive word RT–neutral word RT. *The ICC value was negative due to a negative average covariance among data collected at baseline and retest, which violated reliability model assumptions.

## Discussion

The goal of the current study was to test the psychometric properties of the suicide stroop task. Consistent with previous research (Wilson et al., 2019), the mean RTs for all valence words demonstrated good internal reliability. However, the suicide stroop task performance lacked concurrent validity, as the suicide stroop task did not reveal suicide-related attentional biases among current suicide ideators. We also found that the suicide stroop task performance was not significantly associated with the severity of suicidal ideation, depression, hopelessness, nor anhedonia, which indicated a lack of concurrent validity for the task as well. Additionally, the interference scores of all stimuli showed poor test–retest consistency, whereas other self-report measures (i.e., BSSI, PHQ-9, BHS-4, and SHAPS) showed moderate-to-good test–retest consistency. Thus, the results of this study did not support the use of the suicide stroop task on the identification of suicidal risk among Chinese college students.

There might be some reasons for these results. First, the general reaction time is associated with age-related differences in cognitive ability. Our samples were much younger than those in studies with positive results (Williams and Broadbent, 1986; Becker et al., 1999; Cha et al., 2010). Second, the suicide stroop task might be more sensitive in depressive people with recent suicidal attempts (Chung and Jeglic, 2016), whereas in this study, the majority were not depressive and did not have a recent suicidal attempt. Third, as the suicide stroop task uses manual reaction times (i.e., press a key) in responding to the stimuli as a measure, other paradigms, such as voice and eye movements in responding to the stimuli, might perform better.

This study is limited by a small convenience sample. Among 121 participants, 35 participants had current suicidal ideation, and four participants reported previous suicidal attempts. However, in the community, most people will not report suicidal ideation, and the majority will have never made a serious suicide attempt before. That is the reason why we need more sensitive measures with high accuracy on screening suicidal risk.

Over the past 50 years, there was a surge of research designed to identify the risk factors for suicidal behaviors, and many different theories of suicide have been proposed (Wenzel and Beck, 2008; Franklin et al., 2017). It is still a critical challenge on the identification of suicide risk and the prediction of suicide. We believe it is of great meaning to explore more objective measures or behavior markers related to suicidal behaviors. However, it is crucial to assess the psychometric properties of behavioral measures rigorously as self-report measures before large applications in clinical and community settings.

## Data Availability Statement

The raw data supporting the conclusions of this article will be made available by the authors, without undue reservation.

## Ethics Statement

The studies involving human participants were reviewed and approved by The Affiliated Brain Hospital of Guangzhou Medical University. The patients/participants provided their written informed consent to participate in this study.

## Author Contributions

LN conducted the data analysis and drafted the manuscript. All authors contributed to the study design, provided substantial editorial input in the drafting of the manuscript, and read and approved the final manuscript.

## Conflict of Interest

The authors declare that the research was conducted in the absence of any commercial or financial relationships that could be construed as a potential conflict of interest.
